# Kaposi’s Sarcoma-Associated Herpesvirus-Encoded circRNAs Are Expressed in Infected Tumor Tissues and Are Incorporated into Virions

**DOI:** 10.1128/mBio.03027-19

**Published:** 2020-01-07

**Authors:** Bizunesh Abere, Jinghui Li, Hongzhao Zhou, Tuna Toptan, Patrick S. Moore, Yuan Chang

**Affiliations:** a Hillman Cancer Center, Cancer Virology Program, University of Pittsburgh, Pittsburgh, Pennsylvania, USA; b Department of Microbiology and Molecular Genetics, University of Pittsburgh, Pittsburgh, Pennsylvania, USA; c School of Medicine, Tsinghua University, Beijing, People’s Republic of China; d Department of Pathology, University of Pittsburgh, Pittsburgh, Pennsylvania, USA; University of North Carolina, Chapel Hill

**Keywords:** Kaposi's sarcoma, Kaposi's sarcoma-associated herpesvirus, circular RNA, human herpesvirus, noncoding RNA, tumor virus

## Abstract

Kaposi’s sarcoma-associated herpesvirus (KSHV) has recently been found to generate circular RNAs (circRNAs) from several KSHV genes, most abundantly from K10 (viral interferon regulatory factor 4 [vIRF4]), K7.3, and polyadenylated nuclear (PAN) RNA. To define expression of these circRNAs, KSHV-infected cell lines, patient tissues, and purified virions were examined. KSHV circRNA expression was universally detected in tests of six primary effusion lymphoma (PEL) cell lines but ranged from low-level expression in BC-1 cells dually infected with tightly latent KSHV and Epstein-Barr virus to abundant expression in KSHV-only BCBL-1 cells with spontaneous virus production. Generally, the PAN/K7.3 locus broadly and bidirectionally generated circRNA levels that paralleled the corresponding linear RNA levels. However, RNA corresponding to a particular KSHV circularization site (circ-vIRF4) was minimally induced, despite linear vIRF4 RNA being activated by virus induction. *In situ* hybridization showed abundant circ-vIRF4 in noninduced PEL cells. All three KSHV circRNAs were isolated as nuclease-protected forms from gradient-purified virions collected from BrK.219 cells infected with a KSHV molecular clone. For circ-vIRF4, the fully processed form that is exported to the cytoplasm was incorporated into virus particles but the nuclear, intron-retaining form was not. The half-life of circ-vIRF4 was twice as long as that of its linear counterpart. The KSHV circRNAs could be detected at a higher rate than their corresponding linear counterparts by *in situ* hybridization in archival tissues and by reverse transcription-PCR (RT-PCR) in sera stored for over 25 years. In summary, KSHV circRNAs are expressed in infection-associated diseases, can be regulated depending on virus life cycle, and are incorporated into viral particles for preformed delivery, suggesting a potential function in early infection.

## Abstract (precis)

KSHV has recently been found to encode circRNAs. circRNAs result from back-splicing of an upstream pre-mRNA splice donor exon-intron junction to an acceptor site, generating a covalently closed circle. This study revealed that for one KSHV region, the PAN/K7.3 locus, broadly and bidirectionally generated circRNA levels parallel corresponding linear RNA levels. Another KSHV circularization site (circ-vIRF4), however, showed expression that differed from that of the corresponding linear RNA. All KSHV circRNAs are incorporated into KSHV virions and are potentially expressed as immediate early products in newly infected cells.

## INTRODUCTION

Kaposi’s sarcoma-associated herpesvirus (KSHV)/human herpesvirus 8 (HHV8) causes Kaposi’s sarcoma (KS), an angiogenic neoplasia of endothelial origin (1), and two B-cell lymphoproliferative disorders, primary effusion lymphoma (PEL) (2) and multicentric Castleman disease (MCD) (3). Latent KSHV infection is typical for the virus in tumors and PEL cell lines and is characterized by highly restricted viral gene transcription (4). However, different viral transcription profiles, with less-restricted gene expression and occasional virion production, have been found in different tumor types (5–11).

In addition to >90 KSHV protein-coding mRNAs, most of which are predominantly expressed during full-virus lytic replication, noncoding viral RNAs (ncRNAs) are also functionally important features of the KSHV transcriptome. These KSHV ncRNAs include 12 precursor microRNAs (pre-miRNAs) that give rise to a total of 25 mature miRNAs (12–14). These viral miRNAs are expressed during latency (15, 16) and regulate host and viral gene expression to promote immune evasion, viral persistence, and disease progression (17–21).

A number of KSHV long noncoding RNAs (lncRNAs) (22–26) from intergenic and noncoding regions of the viral genome are also expressed—most abundantly during lytic replication or after *de novo* infection (4, 6, 27–29). Most KSHV lncRNAs other than PAN run antisense to known open reading frames (ORFs). Notable among these are the antisense-to-latency transcript (ALT), which is transcribed antisense to the major viral latency locus; T3.0 and T1.2, which are oriented opposite to replication and transcription activator (RTA/ORF50); and K7.3, which runs antisense to PAN (12, 22, 23, 27, 30).

circRNAs constitute a class of 3′-to-5′ covalently closed, cyclized RNAs derived through back-splicing (BS) of a pre-mRNA such that a donor splice junction (SJ) ligates to an upstream acceptor site (31). circRNAs thus lack a 5′ cap or 3′ poly(A) tail (31, 32). circRNAs have been found to act as miRNA sponges (33, 34), to sequester RNA-binding proteins (35–37), and to regulate isogenic transcription and splicing (31, 35, 38, 39) and may generate protein products through internal ribosome entry site (IRES)-driven or m6A-driven 5′-cap-independent translation (40–42). Recent studies also suggested that cellular circRNAs modulate innate immune responses (43–45). circRNAs are resistant to exonucleolytic decay and therefore have long half-lives compared to linear transcripts from the same gene (31, 46). Some cellular circRNAs have been shown to be abundant in cancer tissues and liquid biopsy specimens and might be useful biomarkers of disease progression or prognosis (47, 48).

KSHV encodes circRNAs from the K10 locus (circ-viral interferon regulatory factor 4 [circ-vIRF4]) and from the PAN and K7.3 loci (49–51). One of the two circ-vIRF4 RNA molecules exhibits intron retention (IR) (50) of the conserved intron that is spliced from the linear vIRF4 mRNA transcript. In addition to circ-vIRF4, a cluster of multiple, bidirectional KSHV circRNAs that do not correspond to known mRNA splice junctions are expressed from the PAN/7.3 locus (49). Each PAN/K7.3 circRNA species is individually of low abundance, but, *en masse*, the species represent a major portion of KSHV-encoded circRNAs. Additional low-abundance KSHV circRNAs have been described as originating from ORF4, ORF6, ORF21, ORF22, ORF34, ORF35, ORF36, ORF37, ORF61, and ORF62 loci in *de novo* infections of endothelial cells (51). We focus on the three KSHV circRNAs most abundantly identified in sequencing of naturally infected PEL cells.

In this study, we characterized the expression profile of KSHV-encoded circRNAs in a panel of PEL cell lines, primary KSHV-associated tumor tissues, and patient-derived liquid biopsy specimens. We found KSHV circRNAs to be ubiquitously but differentially expressed in PEL cell lines. They are incorporated into KSHV virion particles produced from BJAB-rKSHV.219 cells, suggesting a function for viral circRNAs at the initial steps of primary infection that operates in a fashion similar to that seen with the tegument proteins which are involved in innate immune modulation for successful establishment of KSHV-infection (52). Consistent with the predominantly cytoplasmic nature of KSHV virion tegument and envelope acquisition, only a cytoplasmic form of circ-vIRF4 is packaged into the viral particle. KSHV circRNAs are detected at a higher rate than the linear latency-associated nuclear antigen (LANA) transcript in primary KS biopsy specimens as well as in patient-derived sera and plasma; however, viral circRNA detection is highly susceptible to RNA degradation during tissue and liquid biopsy processing. Nevertheless, RNA decay analysis revealed that the rate of turnover of KSHV circ-vIRF4 is twice that of linear vIRF4 as revealed by RNA polymerase (Pol) II inhibition.

## RESULTS

### KSHV-encoded circRNAs are ubiquitously but differentially expressed in PEL cells.

Expression profiles of KSHV-encoded circRNAs were assessed in PEL tumor-derived cell lines infected with KSHV alone (BCP1, BC3, and BCBL1) or dually infected with KSHV and Epstein-Barr virus (EBV) (BC1, JSC1, and BBG1), as well as in BJAB-rKSHV.219, a BJAB cell line stably infected with a recombinant virus (rKSHV.219). BJAB cells negative for KSHV and EBV were used as a negative control. Reverse transcription-PCR (RT-PCR) was performed using junction-spanning, divergent primer (DP) pairs to detect circRNAs or conventional convergent primers (CP) to detect corresponding linear viral transcripts. Oppositely stranded circPAN and circK7.3 RNAs are generated from both overlapping and nonoverlapping transcripts (49), and no individual divergent primer pairs can be designed to detect all KSHV circPAN and circK7.3. Instead, we designed one divergent primer pair each for circPAN and circK7.3 that can detect the majority of the circRNA species.

**FIG 1:**
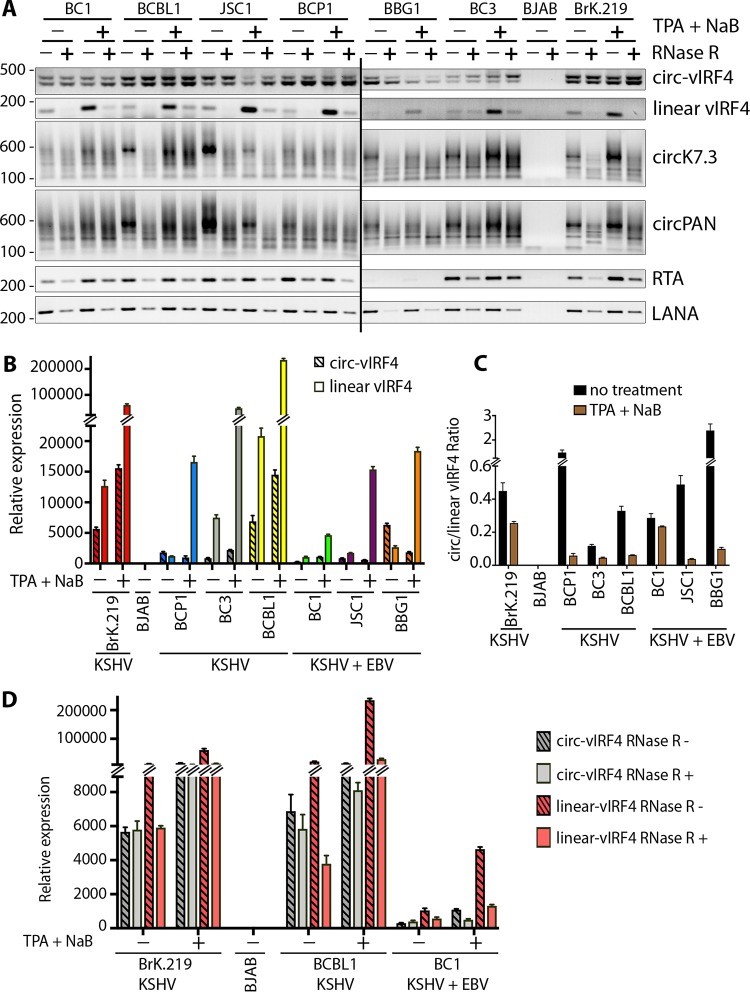
Expression profile of KSHV circRNAs in a panel of PEL cells. Total RNA was extracted from PEL cells with or without induction of the KSHV lytic cycle using a combination of TPA and NaB. (A) RT-PCR was performed on RNase R-treated (+) or untreated (-) samples using divergent primer pairs corresponding to circ-vIRF4, circPANs, or circK7.3, while linear transcripts (vIRF4, LANA, and RTA) were amplified using a pair of convergent primers specific to each gene. (B) Quantitative comparison of circular and linear vIRF4 expression in cDNA from RNase R (-) samples processed as described for panel A using a TaqMan-based qPCR with a probe spanning circ-vIRF4 junction and a probe corresponding to the 3′ end of linear vIRF4, outside the circRNA coding sequence. (C) Ratio of circ-vIRF4 expression to linear vIRF4 expression in the experiment described for panel B. (D) Comparison of circ-vIRF4 expression and linear vIRF4 expression in the presence or absence of RNase R treatment. *C_T_* values were normalized with the RNase P *C_T_* value to calculate delta *C_T_*, and all samples were normalized to BJAB cells to calculate delta-delta *C_T_* (relative expression) values. Bar graphs in panels B, C, and D represent means ± standard deviations (SD) of results from three replicates.

Transcripts of all three KSHV-encoded circRNAs (circ-vIRF4, circPAN, and circK7.3) as well as linear vIRF4 transcripts were found to be expressed across all tested PEL cell lines regardless of KSHV replication status (Fig. 1A). Reactivation of KSHV lytic replication by treatment with 20 ng/ml tetradecanoyl phorbol acetate (TPA) and 3 mM sodium butyrate (NaB) was confirmed by expression of the master lytic switch RTA gene (ORF50) transcript as well as by increased levels of linear vIRF4 (K10). In comparison, linear LANA (ORF73) transcript levels were not affected by replication induction (Fig. 1A). As expected, levels of linear RTA, vIRF4, and LANA RNAs were generally diminished by treatment with RNase R (4 units [U]). In contrast, levels of KSHV circRNAs were stable or were even increased by RNase R treatment, presumably due to reduced PCR template competition. No obvious systematic differences in KSHV circRNA expression levels were found in the KSHV-only PEL cell lines compared to those having EBV coinfection.

To examine KSHV circRNA expression in greater detail, we focused on the circ-vIRF4 transcript. Quantitative RT-PCR performed using the same set of samples as those represented in Fig. 1A together with TaqMan probes corresponding to the circ-vIRF4 back-splice junction (BSJ) and the 3′ end of linear vIRF4 transcript showed differing levels of circ-vIRF4 and linear vIRF4 expression across uninduced PEL cells (Fig. 1B). BCBL1 cells, which show high levels of spontaneous KSHV replication (25), and BJAB-rKSHV.219 cells infected *in vitro* both expressed high levels of circ-vIRF4 and linear vIRF4. In contrast, BC1 cells, which have a 33-kb genomic duplication (53), are tightly latent (25) and showed the lowest levels of circ-vIRF4 and linear vIRF4 expression (Fig. 1B). While most cell lines preferentially transcribed more linear than circular vIRF4 RNA (assuming similar transcript reverse transcription efficiencies), that pattern was reversed for the BCP-1 and BBG1 cells, in which circ-vIRF4 transcripts were more abundant than the corresponding linear transcripts (Fig. 1B and C). This is consistent with previous findings showing that most but not all cellular circRNAs are less abundant than the corresponding linear transcripts (46).

While induction of the KSHV lytic cycle markedly increased the numbers of circPAN and circK7.3 transcripts (Fig. 1A) as well as the linear vIRF4 mRNA levels (Fig. 1A and B) in all cell lines examined, circ-vIRF4 transcription was activated only marginally or was even reduced (Fig. 1A and B). This is more clearly shown in Fig. 1C; the ratio of circular transcripts to linear transcripts from the K10 locus was reduced for all cell lines, suggesting independent mechanisms for regulation of circ-vIRF4 and linear vIRF4 RNA transcriptional biogenesis. Alternatively, it is possible that circ-vIRF4s are induced by reactivation but to a lesser extent than the linear vIRF4 mRNA.

Quantitative analysis of circ-vIRF4 and linear vIRF4 levels in RNase R-treated versus untreated control samples revealed that circ-vIRF4 was resistant to the exonuclease treatment in the unreactivated cells (Fig. 1D; see also Fig. 1A). However, similar analysis of RNA from reactivated cells showed an increase in RNase R sensitivity of circ-vIRF4 whereas linear vIRF4 levels decreased dramatically in response to RNase R treatment regardless of the KSHV reactivation status (Fig. 1D; see also Fig. 1A, B, and Fig. S1).

10.1128/mBio.03027-19.1 FIG S1 Expression profile of KSHV circRNAs in a panel of PEL cells. Total RNA was extracted from PEL cells with or without induction of the KSHV lytic cycle using a combination of TPA and NaB. RT-PCR was performed on samples treated with RNase R (+) or left untreated (-) by the use of divergent primer pairs corresponding to circ-vIRF4, convergent primers corresponding to linear vIRF4, a probe spanning the circ-vIRF4 junction, and a probe corresponding to the 3′ end of linear vIRF4, outside the circRNA coding sequence. (A and B) Comparison of levels of circ-vIRF4 (A) or linear vIRF4 (B) expression in the presence or absence of RNase R treatment. *C_T_* values were normalized to the RNase P *C_T_* value to calculate delta *C_T_* values, and all samples were normalized to BJAB cells to calculate delta-delta *C_T_* (relative expression) values. The bar graphs shown in panels A and B represent means ± standard deviations (SD) of results from three replicates. Download FIG S1, TIF file, 1.8 MB. Copyright © 2020 Abere et al. 2020 Abere et al. This content is distributed under the terms of the Creative Commons Attribution 4.0 International license.

### Virally encoded circRNAs are packaged into KSHV particles.

**FIG 2:**
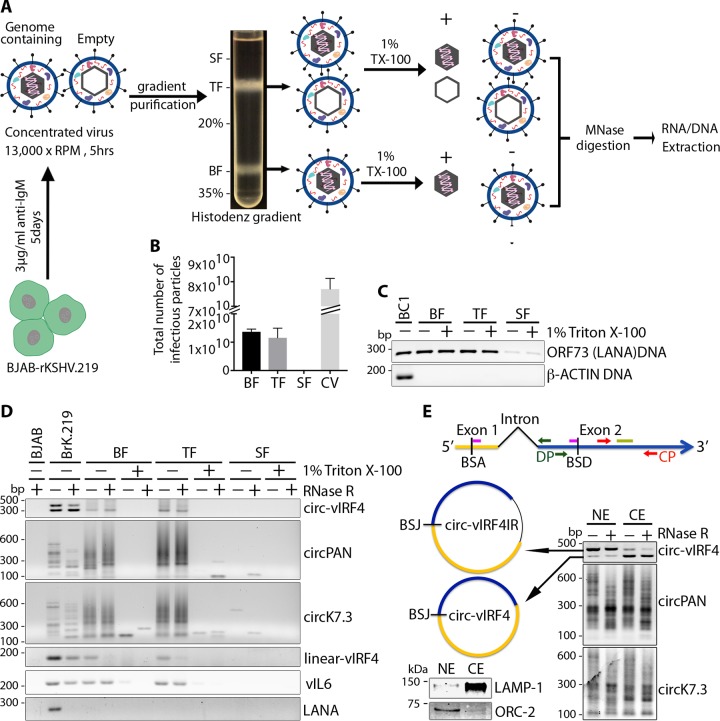
KSHV circRNAs packaged into viral particle. A recombinant KSHV virus was produced from BJAB-rKSHV.219 cells and purified on a 20% to 35% Histodenz gradient. Histodenz fractions (BF, bottom fraction; TF, top fraction; SF, supernatant fraction) were treated with 1% Triton X-100 or left untreated. (A) Experimental workflow. (B) Total numbers of infectious KSHV particles from each Histodenz fraction and the unpurified crude virus (CV) preparation. Bar graphs in panel B show means ± SD of results from three replicates. (C) LANA/ORF73 PCR to detect KSHV genome in DNA extracted from the three different Histodenz fractions with and without Triton X-100 treatment. Genomic DNA from BC1 cells was used as a positive control. (D) RT-PCR detection of KSHV circRNA (circ-vIRF4, circPAN, and circK7.3) and linear RNA (vIRF4) in Histodenz fractions with and without detergent treatment. Linear viral transcript K2/vIL6 was used as a positive control and was packaged into viral particles, while LANA/ORF73 RT-PCR served as a negative control. (E) (Right panel) Nuclear/cytoplasmic fractionation in BC1 cells and KSHV circRNA detection by RT-PCR using divergent primers. (Left panel) Schematic representation of the two differentially localized circ-vIRF4 forms (dark green arrows, DP primers; pink bars, junction-spanning TaqMan probe; red arrows, CP primers; light green bar, TaqMan probe) (top) and a Western blotting control for the fractionation assay using a nuclear marker (ORC-2) and a cytoplasmic marker (LAMP-1) (bottom). NE, nuclear extraction; CE, cytoplasmic extraction. Results are representative of at least 3 independent experiments.

To determine whether KSHV-encoded circRNAs are packaged into virions, KSHV virions were produced from BJAB-rKSHV.219 cells induced with anti-human IgM antibody and purified through a 20% to 35% Histodenz gradient as schematically shown in Fig. 2A. The rKSHV.219 molecular clone encodes a green fluorescence protein (GFP) under the control of a human elongation factor 1-α promoter from a red fluorescent protein (RFP)/GFP/PURO expression cassette inserted between ORFK9 and ORF57 (54), allowing measurement of infectious activity by fluorescence microscopy.

A fast-sedimenting bottom fraction (BF) band containing mainly virions with encapsidated viral genome, a top fraction (TF) band composed of both genome-containing and empty viral particles (55, 56), and the supernatant containing no infectious particles were collected and analyzed (Fig. 2A). In this protocol, the amount of encapsidated, genome-containing virions in the TF is proportional to the sedimentation time. The infectious particles from these three fractions were titrated on HEK-293 cells. The bottom and top band fractions contained similar amounts of infectious viral particles as measured by the number of GFP-positive HEK-293 cells (Fig. 2B). The bar graphs shown in Fig. 2B indicate the absolute number of infectious KSHV particles from each fraction. The amount of mature virions in the top fraction is consistent with the relatively short sedimentation time used. In contrast, the amount of infectious viral particles in the supernatant fraction was negligible. The three fractions were left untreated or treated with 1% Triton X-100 and subjected to micrococcal nuclease (MNase) digestion to remove any unprotected RNA or DNA outside the viral particle.

KSHV genomic DNA was detected by LANA/ORF73 PCR in DNA extracted from both Triton X-100-treated and untreated samples from the bottom and top fractions, but not in the supernatant fraction, indicating the integrity of KSHV capsid after detergent treatment (Fig. 2C) (55). RT-PCR analysis of RNA extracted from these fractions using divergent primers detected all three KSHV-encoded circRNAs (circ-vIRF4, circK7.3, and circPAN) (Fig. 2D) in virion preparations from the samples not treated with Triton X-100 but not in those from treated samples, indicating that circRNAs are likely to be present in the tegument of viral particles protected from MNase-mediated degradation (Fig. 2C). Consistent with a previous report (57), we also detected viral linear RNA vIL6/K2 but not LANA/ORF73 in the KSHV virion gradient fractions.

### Only the cytoplasmic form of circ-vIRF4 is incorporated into KSHV virions.

One of the two circ-vIRF4 RNA molecules has intron retention (IR) (50) (Fig. 1A) of the conserved intron that is spliced from the linear vIRF4 mRNA transcript. Nuclear cytoplasmic fractionation of BC1 cells showed differential subcellular localization of the two circ-vIRF4 forms (Fig. 2E, upper-left panel): the larger intron-containing circ-vIRF4.IR was retained in the nucleus whereas the smaller, processed, exon-only circ-vIRF4 was exported to the cytoplasm (Fig. 2E, right panel). Sequencing of the two PCR products for circ-vIRF4 confirmed that the top band (nuclear form) contained the depicted intron whereas the bottom band did not contain the intron. circPANs and circK7.3s were found in both cellular compartments (Fig. 2E, right panel). The quality of fractionation was monitored by Western blot analysis of cytoplasmic (LAMP-1) and nuclear (ORC-2) marker proteins (Fig. 2E, bottom-left panel). Consistent with the mainly cytoplasmic nature of tegument acquisition and envelopment of KSHV virions (52, 58), only the cytosolic intron-less circ-vIRF4 (Fig. 2E) was incorporated into KSHV particles (Fig. 2D). circ-vIRF4.IR was excluded from virus particles. Due to the number of circRNAs from both the PAN and K7.3 loci, we could not assign these specific circRNAs to particular subcellular compartments; therefore, we were unable to determine whether only cytoplasmic forms of these circRNAs were packaged into viral particles (Fig. 2D). Nevertheless, for circK7.3, there appeared to be increased accumulation of smaller circular forms in the cytoplasmic fraction (Fig. 2E).

### KSHV circPANs are inducible whereas circ-vIRF4 is constitutively expressed in KSHV-infected B cells *in situ*.

**FIG 3:**
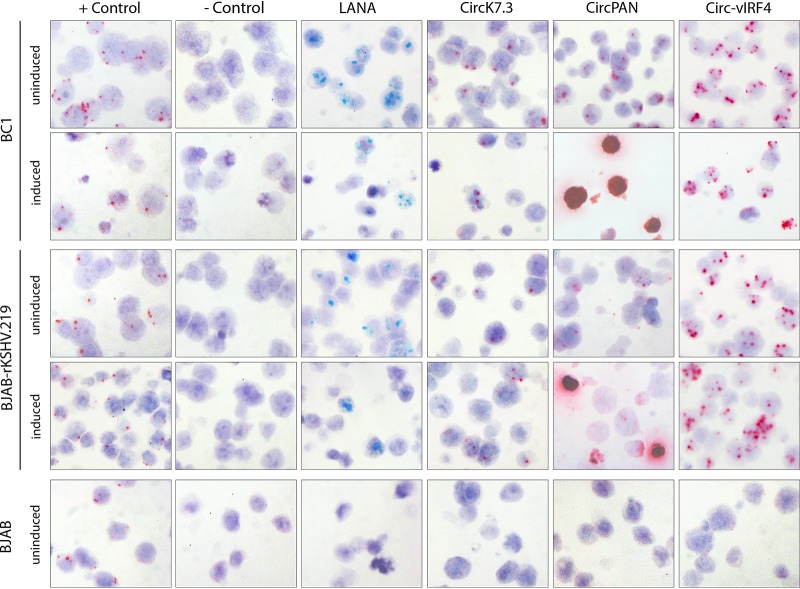
BaseScope RNA ISH detection of KSHV circRNAs in PEL cells. Representative images from induced/uninduced PEL cells are shown. Uninduced BJAB cells were used as a KSHV negative control. Green signals show staining for linear RNA (LANA), while red signal represents detection of the represented KSHV circRNAs or the cellular control POLR2A. For the positive-control slide, only red signal (POLR2A) was developed, so no green signal (PPIB) is visible. Original magnification, ×40.

BaseScope *in situ* hybridization (ISH) probes against the unique KSHV circRNA back-splice junctions were applied to HistoGel-embedded KSHV-infected BC-1, BJAB-rKSHV.219, and KSHV-infection-negative BJAB cell pellets. This was performed using a single probe against the circ-vIRF4 BSJ, three probes to hybridize to the most abundant circPAN BSJs, and three probes against circK7.3 BSJs. KSHV circRNA hybridization results were compared to those obtained using three side-by-side probes (3zz) targeting LANA RNA (see Materials and Methods; see also Table 6 for the list of BaseScope probes used). Formalin-fixed, paraffin-embedded (FFPE) cell pellets were arrayed in a single block to minimize slide-to-slide staining variations (see Materials and Methods for the induction protocol for the various cell lines). Quality control was performed using probes corresponding to human housekeeping genes PPIB and POLR2A to ensure RNA integrity in all cell pellets (Fig. 3, “+ Control” column). Using the bacterial DapB probe as a negative control, none of the pellets showed positive signals in cells (Fig. 3, “- Control” column). BJAB cells were negative for all KSHV linear and circular RNA probes. The linear LANA RNA probe showed similar staining results in the BC-1 and BJAB-rKSHV.219 cells, with no significant changes in the average number of positive-scoring signal dots per cell or in the percentage of positive-scoring cells upon induction compared to no-treatment conditions. Similar results were seen with the circ-vIRF4 and circK7.3 probes. In contrast, the circPAN probe showed a massive increase in signal under induction conditions that obliterated all hematoxylin counterstaining in the affected cells. Of note, the circ-vIRF4 probe showed strong and multiple dot signals in virtually every cell, and although the avidity of a probe with respect to its target can vary depending on the probe, it is clear that circ-vIRF4 BSJs were abundant in the uninduced cells and showed no further increase in abundance upon induction.

### KSHV circRNAs are present in KSHV-associated primary tumors and patient sera.

RT-PCR detection of viral circRNAs in freshly frozen (FF) or optimal-cutting-temperature (OCT) embedded KS tissue samples showed that 14/17 (82.4%) samples were positive for circ-vIRF4 expression and 5/17 (29.4%) positive for circPAN/K7.3 using a divergent primer pair that can detect most circRNAs in both PAN RNA and the anti-sense K7.3 region (Table 1). Considering all three circRNAs together, the detection rate rose to 16/17 (94.1%) compared to only 14/17 samples positive for the linear LANA transcript.

**TABLE 1:** KSHV circRNA detection in KS tissues and sera by RT-PCR

KSHV transcript	No. (%) of samples^*a*^	
	FFT/OFT (*n* = 17)	Serum (*n* = 10)
circ-vIRF4	14 (82.4)	4 (40)
circPAN/K7.3	5 (29.4)	3 (30)
circPAN/K7.3 + circ-vIRF4	16 (94.1)	5 (50)
LANA	14 (82.4)	NA

a FFT, freshly frozen tissue; OFT, OCT embedded tissue; *n*, number of tumors; NA, not applicable.

BaseScope probes (see Table 6) against the unique KSHV circRNA back-splice junctions were used on archival formalin-fixed, paraffin-embedded (FFPE) KS tissues. To assess KSHV circRNA ISH, tissues were first assessed for RNA integrity and lack of nonspecific hybridization. A total of 181 archival KS skin, oral, or visceral tumors were screened. Of these, 92 were considered valid for analysis based on the tissue RNA quality determined by confirmation of both (i) positive staining corresponding to a human housekeeping gene (*PPIB* or *POLR2A* 1zz probes) and (ii) low levels of nonspecific background staining (fewer than 10 positive-scoring [+] dots per tissue for a negative-control bacterial gene [DapB 1zz probe]) (Table 2).

**TABLE 2:** KS tumors used for KSHV circRNA BaseScope ISH detection^*a*^

Tumor type	No. of cases			
	Total	False positive	No tissue	Valid
KS	181	33	56	92
Non-KS skin/cancer	24	11	2	11

a All tissues tested were obtained arrayed onto glass slides. The KS lesions were from the AIDS and Cancer Specimen Resource (ACSR), and the non-KS skin/cancer controls were from US Biomax (Derwood, MD) and included 6 squamous cell carcinoma tissue samples, 6 basal cell carcinoma tissue samples, 2 syringocarcinoma tissue samples, 4 malignant melanoma tissue samples, 2 dermatofibroma protuberans tissue samples, and 4 cancer adjacent skin tissue samples.

**FIG 4:**
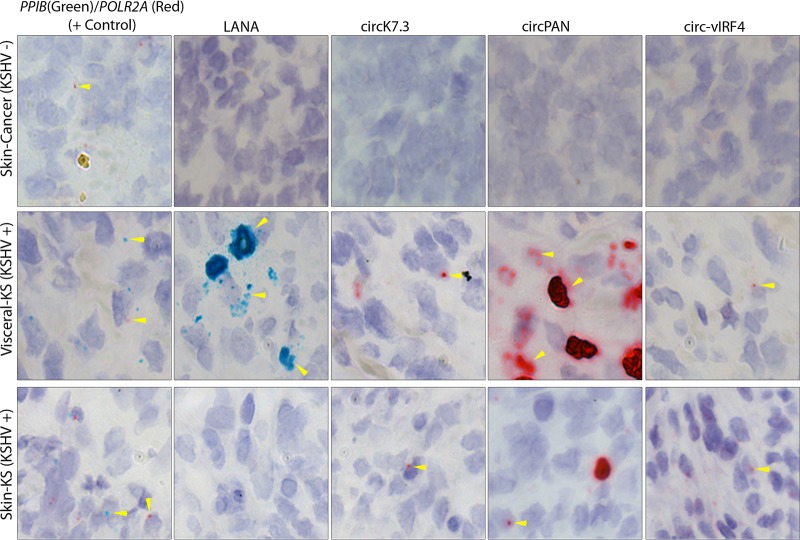
BaseScope RNA ISH detection of KSHV circRNAs in primary KS tumor tissues. Representative images from a visceral KS tumor tissue and a skin KS tumor tissue are shown. A malignant melanoma, non-KS skin cancer tissue, was used as negative control for the KS lesions. Green signals show staining for linear RNAs (control PPIB or LANA), while red signal represents detection of the represented KSHV circRNAs or the cellular control POLR2A. Arrowheads show positive staining from circular RNA or linear RNA. Original magnification, ×40.

A total of 61 (66.3%) of 92 valid KS tissue biopsy specimens were positive for KSHV circRNAs, including 32 of 92 (34.8%) for circ-vIRF4, 49 of 92 (53.3%) for circPAN, and 28 of 92 (30.4%) for circK7.3. By comparison, 35 of 92 (38%) were positive for LANA mRNA (Table 3). Representative images of KS tumors are shown (Fig. 4). The variations in the levels of KSHV circRNA expression in these tissues can be partially attributed to differences in RNA quality due to tissue processing and source; we also cannot rule out the possibility of differential expression at this point.

**TABLE 3:** KSHV circRNA detection in KS tumors by BaseScope ISH

KSHV transcript	No. (%) of KS tumors (*n* = 92)
circPAN	49 (53.3)
circK7.3	28 (30.4)
circ-vIRF4	32 (34.8)
circPAN + circK7.3 + circ-vIRF4	61 (66.3)
LANA	35 (38.0)

Next, we examined the expression of KSHV circRNAs in KS patient-derived sera. Of 10 sera collected between 1995 and 1999, 4 (40%) were positive for circ-vIRF4 and 3 (30%) were positive for circPAN/K7.3 (Table 1). Fifty percent of these samples were positive for at least one KSHV circRNA (Table 1), while 100% of the freshly prepared plasma and serum samples from four KS patients were positive for KSHV circRNAs.

### KSHV circ-vIRF4 is more stable than its linear transcript.

**FIG 5:**
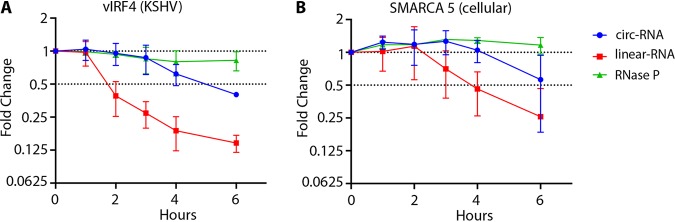
Circular versus linear vIRF4 RNA decay rates. BCBL1 cells were treated with 2 μM flavopiridol, 1 × 10^7^ cells were collected every hour for 6 h, and qRT-PCR was performed using a TaqMan probe spanning the circRNA junction and a probe corresponding to the 3′ end of the linear transcript outside the circRNA coding sequence. (A) vIRF4. (B) A cellular control (SMARCA5). RNase P was used as a control that was not affected by RNA Pol II inhibition. Error bars show means ± SD of results from three replicates from 3 independent experiments.

To analyze circ-vIRF4 turnover, BCBL1 cells were treated with the RNA polymerase II inhibitor flavopiridol (2 μM) and RNA decay rates were determined by quantitative RT-PCR using TaqMan probes specific to the BSJ or to the linear RNA sequence outside the circRNA coding region of circ-vIRF4. Probes were similarly designed for SMARCA5, a cellular gene control that produces both circular and linear RNAs. RNaseP linear RNA, transcribed by RNA pol III and therefore not susceptible to flavopiridol inhibition, was used for treatment control. Consistent with previous reports from studies comparing cellular circRNAs to their linear mRNA counterparts (31, 46), circ-vIRF4, with a calculated half-life of ∼5 h, displayed a lower turnover rate than vIRF4 mRNA in the same samples, which had a half-life of ∼2 h (Fig. 5A). Similar values were found for cellular circSMARCA5, with a half-life of ∼6 h, and linear SMARCA5 mRNA, with a half-life of ∼4 (Fig. 5B). The levels of RNase P RNA were unchanged throughout the 6-h flavopirodol treatment time course.

## DISCUSSION

KSHV-encoded circRNAs represent a newly described category of viral RNA molecules (49, 51); however, their function in the context of virus biology and their contribution to viral pathogenesis remain to be defined. To begin to investigate this, we characterized the expression profiles of circ-vIRF4, circPANs, and circK7.3s in a panel of PEL cell lines; measured whether these cyclized RNA molecules are packaged in KSHV virus particles; determined their stability; and assayed for their presence in patient-derived KSHV-associated tumor tissues/sera to assess their usefulness as a clinical markers of infection.

KSHV circRNAs (circ-vIRF4, circPAN, and circK7.3) are ubiquitously but differentially expressed across PEL cell lines (Fig. 1). Expression of circPANs and circK7.3s is induced upon KSHV lytic replication, which mirrors that of their linear lncRNA counterparts, PAN and K7.3. The lncRNA PAN is the best studied and has been functionally determined to interact with transcriptional and epigenetic modifiers to regulate host and viral gene expression (59–61). The 1.08-kb PAN 3′ region is polyadenylated but does not associate with polyribosomes and contains no known introns (24). The lack of splicing in PAN transcripts suggests a novel mechanism for circular RNA formation from this viral precursor RNA. While PAN is detected at low levels in latent PEL cells (4), it becomes the most abundant viral transcript during lytic KSHV replication. Knockdown studies revealed that PAN is essential to efficient late lytic KSHV gene expression (62). PAN mainly localizes to the nucleus but can also be found in the cytoplasm and packaged into KSHV virions, where it may be active in newly infected cells as an immediate early RNA (57, 60). The K7.3 transcript arising from the strand opposite PAN (25, 30), has unknown function(s). Like PAN, K7.3 RNA is induced during virus replication, albeit at much lower abundance, and while it is assumed to be noncoding, this has not been formally examined (25).

In contrast to circPAN, circ-vIRF4 levels show no consistent changes upon induction of lytic replication, suggesting that its biogenesis is not directly regulated by transcription activity of the vIRF4 operon. KSHV vIRF4 represents one of four ORFs (vIRF1 to vIRF4) encoded by a cluster of interferon regulatory factor (IRF) homologs in the KSHV genome (53). The vIRFs inhibit their cellular counterparts and are also involved in cell cycle regulation and oncogenic transformation (63, 64). Expression of KSHV vIRF1, vIRF2, and vIRF4 is inducible, whereas KSHV vIRF3, which encodes a nuclear protein called LANA2, is latently expressed in all KSHV-infected B cells (65). LANA2 is detected in PEL and MCD (4, 65) but not in KS. Although expression of vIRF4 RNA and protein can be induced by TPA treatment in PEL cells (10, 66), circ-vIRF4 is detectable in unstimulated cells. However, unlike the results seen with its cognate linear transcript, circ-vIRF4 expression as evaluated by RT-PCR showed only a modest increase, or even a decrease, during lytic replication, which is consistent with latency expression (Fig. 1A to C). Of note, by BaseScope *in situ* hybridization, the circ-vIRF4 probe showed strong and multiple dot signals in virtually every KSHV-infected PEL cell (Fig. 3). Expression of circ-vIRF4 biomolecules was present at significant levels in uninduced cells and showed no further increase upon induction. The latent profile of circ-vIRF4 expression suggests a possible regulatory role for this circRNA. In most of the PEL cells, the level of circ-vIRF4 expression was lower than that seen with its linear counterpart, which is consistent with previous reports indicating that the majority of cellular circRNAs are expressed at lower levels than their cognate linear counterparts (46). However, among the six PEL cell lines tested by RT-PCR, the BBG1 and BCP1 cells showed higher circ-vIRF4 expression than linear vIRF4 expression; also, circ-vIRF4 expression decreased after lytic induction in contrast to the linear transcript. This variation could be attributed to the differences in the origins of the cells (the BBG1 cell line was derived from a cutaneous B-cell lymphoma) or could represent a result of cell culture adaptation but could not be attributed to the number of KSHV genome copies, since BBG1 is estimated to have ∼200 copies/cell and BCP1 contains ∼150 copies/cell (67, 68). Further experimentation on the kinetics of these circRNAs at different time points during KSHV lytic induction would shed more light on their biogenesis and potential function.

PAN RNA accumulates in the nucleus of infected cells during KSHV lytic replication, but it is also present in unstimulated KSHV-infected cells (6, 9). A significant amount of PAN is localized to the cytoplasm and incorporated into KSHV virions (57, 60). In addition to PAN, KSHV ORF59, ORF58, ORF54, ORF17, K2, K4, K5IR, K6, K7, K8.1, and K12 RNAs have been found packaged into KSHV virions in which they are directly released into newly infected cells and are presumed to act as immediate early genes early in infection (57). To study whether KSHV circRNAs could be packaged into budding virions, recombinant rKSHV.219 was produced from stably infected BJAB cells using an anti-IgM antibody to cross-link the B-cell receptor (BCR) (69, 70). Similarly to PAN, circPANs, circK7.3s, and circ-vIRF4 were found packaged in nuclease-resistant viral particles (Fig. 2). PAN RNA has already been implicated in modulation of the cellular immune response as well as viral gene expression to facilitate KSHV infection (59, 60, 71–74). Similarly, viral microRNAs and proteins packaged in KSHV virions modulate innate immune functions during primary infection (52). Experimentation will be needed to determine whether virion-packaged KSHV circRNAs might have similar roles.

Two forms of circ-vIRF4 are detectable in infected PEL cells: a larger form (632 nucleotides [nt]) generated through intron retention of the canonical linear vIRF4 intron (102 nt) and a smaller form (530 nt) with a naturally occurring intron spliced out (Fig. 2D and E). Our cytoplasmic/nuclear fractionation experiments showed that circ-vIRF4.IR localizes to the nucleus and was not found in virus particles whereas the exon-only form was cytoplasmic and retained in the virion tegument. This is consistent with herpesvirus tegumentation and the envelopment maturation that occurs in the cytoplasmic compartment (52, 58). circPANs and circK7.3s are also packaged into viral particles; however, given the high number and different sizes of circRNAs from these regions, we were unable to determine whether only cytoplasmic forms of these circRNA are incorporated into the KSHV virion.

KSHV-encoded circRNAs can be detected in patient-derived primary tumor tissues *in situ* and in liquid biopsy specimens, even in tissues that had been fixed and stored at room temperature for decades. RNA degradation occurring during tumor tissue fixation/storage as well as liquid biopsy preparation might also lead to underestimation of circRNA levels. Despite this remarkable retention of RNA in archival tissues, it is unlikely that circRNA detection can surpass the sensitivity of KSHV protein or antibody detection.

circRNAs are more stable than the corresponding linear cognate RNAs mainly due to their covalently closed circular nature, which confers resistance to exonucleolytic decay. Estimates of the half-life of cellular circular RNAs range between 19 to 24 h, 2.5 times longer than that of their linear counterparts (31, 32, 46, 75). Consistent with the stability of cellular circular RNAs, circ-vIRF4, with a half-life of 5 h in unreactivated BCBL1 cells, showed a half-life that was double that of its linear transcript (Fig. 5). The stability of circ-vIRF4 relative to that of the corresponding linear mRNA points to the intriguing possibility of differential regulation of RNA biogenesis through circularization.

## MATERIALS AND METHODS

### Cell lines.

KSHV-and-EBV-coinfected BC1 (2) and JSC1 (76) cells; KSHV-positive BCBL1 (77), BC3 (78), and BCP1 (79) cells; and EBV/KSHV-negative BJAB cells (80) were obtained from the American Type Culture Collection (ATCC). The KSHV-and-EBV-coinfected BBG1 cell line (81) was kindly provided by Shou-Jiang Gao (University of Pittsburgh, Pittsburgh, PA, USA), while BJAB-rKSHV.219 cells (69, 70) (BJAB infected with a recombinant KSHV.219 virus [54]) were a generous gift from Thomas F. Schulz (Hannover Medical School, Hannover, Germany). All cell lines were maintained in RPMI 1640 media (Cellgro) supplemented with 10% fetal bovine serum (FBS) (VWR Seradigm). BJAB-rKSHV.219 cells were maintained with 4.2 μg/ml of puromycin selection in RPMI media supplemented with 10% FBS. To induce KSHV lytic reactivation, all PEL cells were treated with 20 ng/ml of TPA and 3 mM NaB for 48 h.

### RNA isolation and DNase and RNase R treatment.

Total RNA was isolated from tumor samples, cell lines, virus preparations, or cellular/virus preparation fractions using TRIzol or TRIzol-LS (Invitrogen) and processed with a Turbo DNA-free kit (catalog no. AM1907; Invitrogen) following the manufacturer’s protocol. A 1-μg volume of DNase-digested RNA was then treated with 4 U of RNase R (catalog no. RNR07250; Lucigen) in 1× RNase R reaction buffer at 37°C for 30 min in the presence of 8 U of RiboLock RNase inhibitor (catalog no. EO0381; Thermo Scientific); for untreated samples, nuclease-free water was added to the reaction mixture in lieu of RNase R. To inactivate the RNase R, all samples were then incubated at 65°C for 20 min.

### Semiquantitative and quantitative RT-PCR.

A 1-μg volume of DNase-digested total RNA that had been either treated with RNase R or left untreated was reverse transcribed with random hexamers by the use of a SuperScript IV first-strand synthesis system (catalog no. 18091; Invitrogen) according to the manufacturer’s protocol. Semiquantitative PCR was then performed using either Q5 high-fidelity DNA polymerase (catalog no. M0491; New England BioLabs/NEB) for circRNA detection or *Taq* DNA polymerase with ThermoPol buffer (catalog no. M0267; NEB) for linear RNA detection, according to the protocols specified for the products.

TaqMan universal master mix II with uracil-N-glycoslyase (UNG) (catalog no. 44400; Applied Biosystems) was used for quantitative PCR (qPCR) analysis of cDNA samples, while an iTaq universal SYBR green one-step kit (catalog no. 172-515; Bio-Rad) was used for direct quantitation of RNA derived from serum and plasma. Threshold cycle (*C_T_*) values were used to calculate the mRNA fold changes according to the delta-delta *C_T_* method. The RNase P *C_T_* value was used as a reference. PCR primers and probes are listed in Tables 4 and 5.

**TABLE 4:** PCR primers

Primer name	Sequence
circ-vIRF4_F (DP)	GCGCTCTCCAAGTATACGTGGCAA
circ-vIRF4 R2 (DP)	CAAATGCATGGTACACCGAATAC

vIRF4 C-term F	GAGTGGCGTTTCTGGAGTAT
vIRF4 C-term R	CTTCGCATACCGCGTCTTA

Linear LANA R	GTTTAGTGTAGAGGGACCTTGGG
Linear LANA F	TCTCCATCTCCTGCATTGCC
Linear RTA/ORF50 F	CACCCCCCGATTCAACAGTC
Linear RTA/ORF50 R	CCTCTCTTTGCTTCTCTGCTTTCG

circPAN divF1 (DP)	CGCCCACTGGTGTATCAGA
circPAN divR1 (DP)	AATCGCAGCTTTTGTTCTGC
circK7.3 divF2 (DP)	ACGGAAAACCTAGCCGAAAG
circK7.3 divR2 (DP)	CGGGTTATTGCATTGGATTC

**TABLE 5:** qPCR primers and probes

Primer/probe name	Sequence or description^*a*^
circ-vIRF4_qPCR_F (DP)	TGGCGATATAACGACTGAACAGAA
circ-vIRF4_qPCR_R (DP)	CAAATGCATGGTACACCGAATACC
circ-vIRF4_BSJ_P	FAM-ATCTACCTCAGCCCCCGCGC-QSY

C-termin-vIRF4_qPCR_F2	ACGGATTCCCAATATTTTGCTC
C-termin-vIRF4_qPCR_R2	GTTCATAGCGGGAATTTGGATT
C-termin-vIRF4_TaqP2	ABY-TCAAGTGGAAGGCTGGTGGTTTGGT-QSY

POP4 (RNase P)	VIC-MGB; Hs00198357_m1 (catalog no. 4331182)

a FAM, 6-carboxyfluorescein.

### Virus production and Histodenz gradient purification.

The rKSHV.219 virus was produced as previously described (69, 82). Briefly, to induce the KSHV lytic cycle, BJAB-rKSHV.219 cells were inoculated at a density of 6 × 10^5^ cells/ml in a total volume of 800 ml culture in the presence of 3 μg/ml of anti-human IgM antibody (catalog no. 2020–01; SouthernBiotech) and grown for 4 to 5 days in Techne spinner flasks (Cole-Parmer GmbH, Wertheim, Germany) with 60 rpm agitation. Culture supernatant was collected after low-speed centrifugation was performed to remove cells and debris and was concentrated by ultracentrifugation at 15,000 rpm for 5 h in a type 19 rotor (Beckman Coulter Inc., Brea, CA, USA). The pelleted virus was resuspended in 1× phosphate-buffered saline (PBS) overnight, layered onto a 20% to 35% Histodenz (D2158; Sigma) gradient, and centrifuged at 71,000 × *g* for 2 h using an SW41Ti rotor (Beckman). The desired fractions were collected, diluted in 1× PBS, and centrifuged at 29,000 × *g* for 2 h using an SW 32 rotor (Beckman). The pellets from each fraction were resuspended in 1× PBS and layered onto the same Histodenz gradient for a second round of purification. Each purified fraction (bottom, top, and supernatant) was then divided in two. One group was treated with 1% Triton X-100, while the other group was left untreated. Both groups of samples were then treated with 15 U/μl of micrococcal nuclease (NEB) in a 500-μl total volume and incubated at 37°C for 20 min. EGTA (5 mM) was added to each sample to stop the reaction, and each was then placed on ice for 30 min. Each sample was further divided into two tubes of 250-μl sample volume, and 750 μl of TRIzol LS reagent was added to each tube for RNA extraction.

The infectious virus titer was determined for each gradient fraction by applying serial dilutions of each preparation on HEK-293 cells as described previously (83). Three days after infection, the number of GFP-positive cells was counted to calculate the KSHV infectious virus titer for each fraction.

### Nuclear/cytoplasmic fractionation.

Nuclear/cytoplasmic fractionation was performed using 1 × 10^7^ BC1 cells with NR-PER nuclear and cytoplasmic extraction reagent (catalog no. 78833; Pierce), according to the manufacturer’s protocol in the presence of RiboLock RNase inhibitor (catalog no. EO0381; Thermo Scientific). A 750-μl volume of TRIzol reagent was added to 250 μl of each fraction for RNA extraction. A 1-μg volume of total RNA from each fraction was used for cDNA synthesis, and the expression level of the indicated circRNAs in each fraction was analyzed by RT-PCR. The quality of the fractionation assay was controlled by immunoblotting for a nuclear marker, ORC-2 (catalog no. 551178; BD Biosciences), and for a cytoplasmic marker, LAMP-1 (eBioH4A3; eBioscience).

### Tumor samples and sera/plasma.

Seventeen pathologically confirmed freshly frozen (FF) or optimal cutting temperature (OCT) embedded KS tissue specimens and 5 KS FFPE tissue microarrays (TMA) (1 visceral tissue microarray, 1 oral tissue microarray, and 3 skin KS tissue microarrays) were obtained from the AIDS and Cancer Specimen Resource (ACSR; San Francisco, CA, USA). Tissues were snap-frozen and stored in liquid nitrogen until use, while the TMAs were used within 2 months of sectioning. Ten archival serum samples and 4 fresh parallel serum samples and plasma samples from patients with AIDS-associated Kaposi’s sarcoma were obtained as by-products of diagnostic or therapeutic procedures performed at the Columbia University College of Physicians and Surgeons and at the University of Pittsburgh Medical Center (UPMC) with the approval of the Institutional Review Board. Specimens were deidentified prior to use in our study.

### Cell pellet preparation.

At 24 h after lytic induction, BC1/BJAB/BJAB-rKSHV.219 cells were harvested and pellets were fixed in 10% neutral buffered formalin and processed with HistoGel (catalog no. HG-4000-012; Thermo Scientific) for routine histology. Formalin-fixed paraffin-embedded cell pellets were then arrayed in a single block to minimized slide-to-slide staining variations. Sections (5 μm) were cut and stained according to the BaseScope protocol described below.

### BaseScope RNA *in situ* hybridization (ISH).

BaseScope RNA ISH was performed using either a BaseScope duplex detection reagent kit (catalog no. 323810) or a BaseScope Detection Reagent v2-RED kit (catalog no. 323910; Advanced Cell Diagnostics) with probes targeting circRNA BSJ or linear RNAs (see Table 6 for the list of probes used). FFPE section slides were baked in a dry oven for 1 h at 60°C, followed by deparaffinization performed with xylene twice for 5 min each time and with 100% ethanol twice for 2 min each time. Slides were then baked for 5 min at 60°C and incubated with RNAScope hydrogen peroxide for 10 min at room temperature followed by two 2-min washes in wash buffer and then with RNAScope 1× target retrieval reagent for 15 min at 98°C. Following target retrieval, slides were transferred into 100% alcohol for 3 min, dried at 60°C, and treated with RNAScope protease IV for 30 min at 40°C. After washing was performed, slides were hybridized with specific BaseScope probes (see Table 6) for 2 h at 40°C in a HybEZ oven (Advanced Cell Diagnostics) followed by amplification comprised of incubations with AMP1 for 30 min at 40°C, AMP2 for 30 min at 40°C, AMP3 for 15 min at 40°C, AMP4 for 30 min at 40°C, AMP5 for 30 min at 40°C, AMP6 for 15 min at 40°C, AMP7 for 30 min at room temperature, and AMP8 for 15 min at room temperature for red signal. For the green signal, after the AMP5 incubation step described above, slides were treated with AMP10 for 15 min at 40°C, AMP11 for 30 min at room temperature, and AMP12 for 15 min at room temperature. Slides were washed twice with 1× wash buffer for 2 min at room temperature between the incubation periods. Chromogenic signals were developed by incubation with either BaseScope Duplex Fast Red or BaseScope Duplex Green solution for 10 min at room temperature in the dark and by counterstaining with 50% Gill’s hematoxylin I staining solution (catalog no. HXGHE1PT; American MasterTech Scientific) for 1 min at room temperature. Slides were dipped in 0.02% ammonia water (221228; Sigma-Aldrich) 2 or 3 times, washed with deionized water, and mounted with VectaMount (64742-48-9; Vector Laboratories).

**TABLE 6:** List of BaseScope detection probes purchased from ACD

Target	BaseScope probe	Part ID^*a*^ or catalog no.
circ-vIRF4-BSJ	BA-V-KSHV-vIRF4-circRNA-C2	715981-C2

circPAN-BSJ	BA-V-HHV8-T1.1-circRNA	719981
	BA-V-HHV8-T1.1-circRNA-O1	719991
	BA-V-HHV8-T1.1-circRNA-O2	720001

circK7.3-BSJ	BA-V-HHV8-K7-sense-circRNA-O1-C2	719951-C2
	BA-V-HHV8-K7-sense-circRNA-O2-C2	719961-C2
	BA-V-HHV8-K7-sense-circRNA-O3-C2	719971-C2

Linear LANA	BA-V-KSHV-LANA-3zz-st	716011

a ID, identifier.

circPAN and circK7.3 were detected using a mixture of three 1zz probes targeting the three most abundant circPAN BSJs as determined by sequencing or using a mixture of three 1zz probes targeting the three most abundant circK7.3 BSJs. circ-vIRF4 was detected with a single 1zz probe for its BSJ, and a single 3zz probe was used to detect linear LANA RNA. Control probes for the human housekeeping genes PPIB and POLR2A and a probe against the bacterial DapB gene were used as positive and negative controls, respectively. Chromogenic signals were visualized with an Olympus AX70 microscope, and the images were acquired using a QImaging QIClick charge-coupled-device (CCD) camera and Q-Capture Pro 7 software and scored as follows: a minus sign (“−”) represents a negative result, and one to four plus signs (“+” to “++++”) represent positive results corresponding to the total number of the dots or clumps (aggregated dots) counted in each 1.5-mm diameter tissue microarray spot. Scoring results are presented as follows: minus sign (“−”), no dots; one plus sign (“+”), 1 to 5 dots and no clumps; two plus signs (“++”), 6 to 10 dots or 1 clump; three plus signs (“+++”), 11 to 30 dots or 2 to 5 clumps; four plus signs (“++++”), >30 dots or > 5 clumps. Background signal for the negative-control probe that targeted bacterial DapB varied greatly depending on source, fixation, and tissue processing of the microarrays. Therefore, as a conservative measure, cases were evaluated only if they (i) were assigned a score of “−” or “+” with the negative-control probe and (ii) were assigned a score of “+” to “++++” with the positive-control probe. Representative images were captured from each group.

### RNA decay analysis of RNA polymerase II inhibition.

BCBL1 cells were inoculated at a density of 2 × 10^6^ cells/ml using fresh medium in the presence of 2 μM flavopiridol, and then 5 ml of cell suspension was divided into aliquots and placed in 15-ml conical tubes. The tubes were loosely capped and incubated at 37°C. Cells were collected at 0, 1, 2, 3, 4, and 6 h after inhibitor treatment by centrifugation at 400 × *g* for 5 min. The cell pellet was then lysed in 1 ml of TRIzol reagent and stored at –80°C until the RNA extraction step. Threshold cycle (*C_T_*) values were used to calculate the mRNA fold changes according to the delta *C_T_* method, and the result obtained at each time point was normalized to the 0-h control within the same group. The *C_T_* value calculated for RNase P was used as a reference to show that the cell numbers were consistent and that global RNA homeostasis was not affected by the presence of flavopiridol.
