# Supplementary figures and images for: Kaposi’s Sarcoma-Associated Herpesvirus-Encoded circRNAs Are Expressed in Infected Tumor Tissues and Are Incorporated into Virions
Source: mBio. 2020 Jan 7;11(1):e03027-19. doi: 10.1128/mBio.03027-19 (PMC6946807; doi:10.1128/mBio.03027-19)

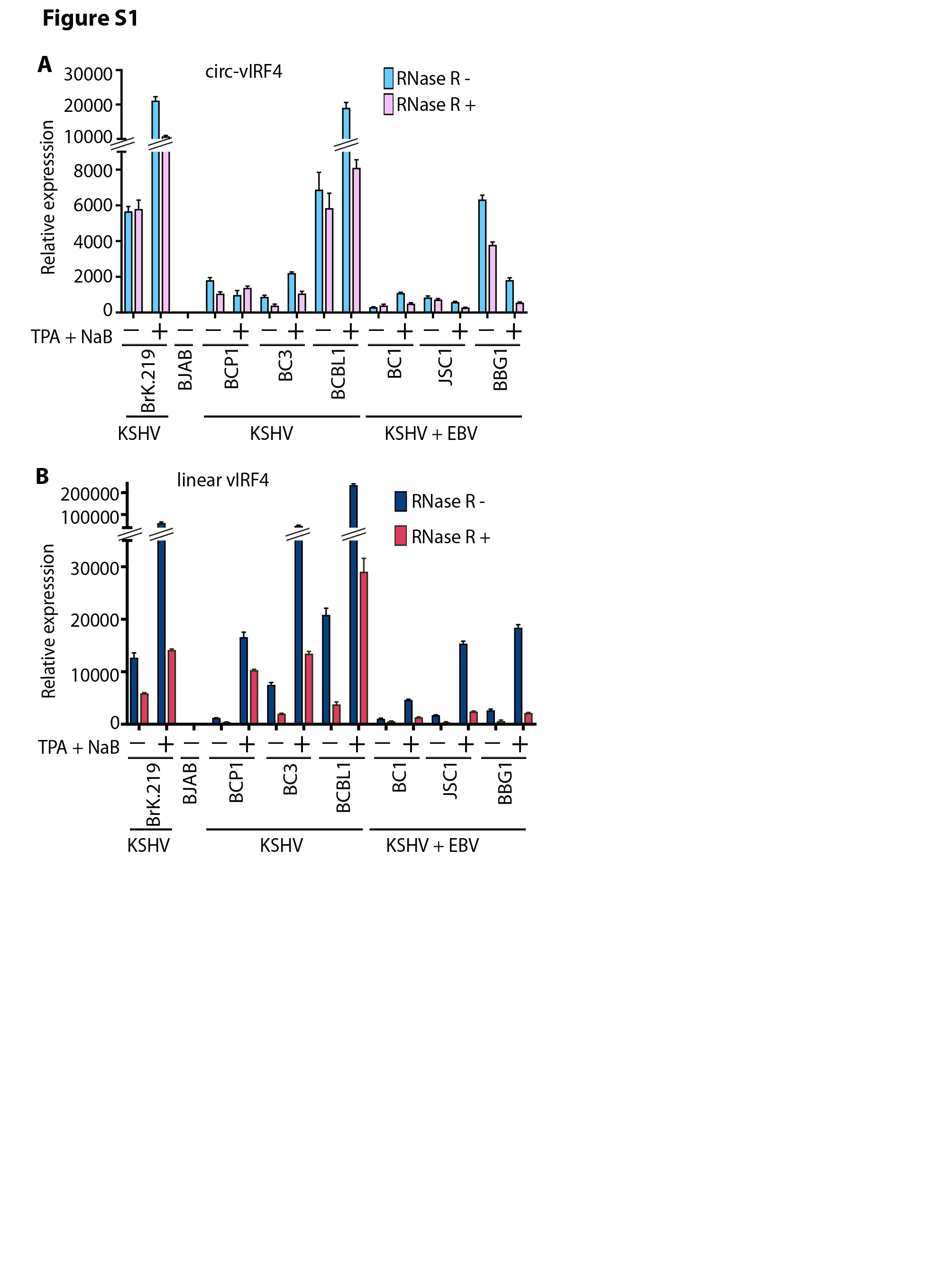

Supplement: FIG S1 [file mBio.03027-19-sf001.tif]
